# Selective activation of TCR-*γδ*^+ ^cells in endemic Burkitt's lymphoma

**DOI:** 10.1186/1475-2875-6-69

**Published:** 2007-05-23

**Authors:** Godfred Futagbi, Jennifer E Welbeck, John Kweku A Tetteh, Lars Hviid, Bartholomew D Akanmori

**Affiliations:** 1 Immunology Department, Noguchi Memorial Institute for Medical Research, University of Ghana, Legon, Ghana; 2 Department of Zoology, University of Ghana, Legon, Ghana; 3 Department of Child Health, Korle-Bu Teaching Hospital, Accra, Ghana; 4 Department of Infectious Diseases and Department of Clinical Microbiology, Copenhagen University Hospital Rigshospitalet, Copenhagen, Denmark and Institute of Medical Microbiology and Immunology, University of Copenhagen, Copenhagen, Denmark

## Abstract

**Background:**

The overlap in geographical distribution of *Plasmodium falciparum *malaria and endemic Burkitt's lymphoma (eBL) – an aggressive Epstein-Barr virus (EBV)-associated B-cell tumour occurring almost exclusively in the tropics – strongly suggests a link between the two diseases. It is suspected that the polyclonal B-cell activation in *P. falciparum *malaria may precipitate a breakdown in homeostatic T-cell control of EBV-immortalized B-cell proliferation. Previous studies have suggested that a particular T-cell subset, characterized by expression of V*δ*1^+ ^*γδ *T-cell receptors, is important for maintaining B-cell homeostasis, both in *P. falciparum*- exposed populations and in individuals subject to polyclonal B-cell activation of other aetiology. The objective of the present study was, therefore, to characterize lymphocyte phenotypes and to investigate possible differences in T-cell subset composition and activation status in *P. falciparum*-exposed Ghanaian children with and without eBL.

**Methods:**

Venous blood samples in heparin from 21 eBL patients (mean age: 7.0 years; range: 3–11 years), referred to the Burkitt's Tumour Centre at Korle-Bu Teaching Hospital, Accra and 15 healthy, age and sex matched children, were stained with fluorescein isothiocyanate (FITC)-, phycoerythrin (PE)-, R-phycoerythrin (RPE)- and RPE-Cy5-conjugated antibodies (CD3, CD4, CD8, CD25, CD69, CD95, HLA-DR, TCR-*γδ*, V*δ*1, V*δ*3, V*γ*9 and B-cells) and acquired on a flow cytometer.

**Results:**

A reduction in the proportion of CD3^+ ^cells in eBL patients, due mainly to perturbations among TCR-*γδ*^+ ^cells was observed. In contrast, the proportions of CD4^+ ^or CD8^+ ^cells were relatively unaffected, as were the mean numbers of peripheral blood mononuclear cells.

**Conclusion:**

Selective changes in numbers and activation status of TCR-*γδ*^+ ^cells occurs in Ghanaian children with eBL, a pattern which is similar to *P. falciparum*-induced changes. The data supports the hypothesis of a regulatory role for V*δ*1^+ ^TcR-*γδ *T-cells in maintaining B-cell homeostasis and provides insights into the pathogenesis of eBL.

## Background

Epstein-Barr virus (EBV) is a ubiquitous herpes virus that can infect and immortalize B-cells. In the vast majority of cases, EBV infection is either subclinical or leads to the self-limiting lymphoproliferative disorder infectious mononucleosis (IM). In both cases, there is an eventual life-long persistence of the virus in the absence of clinical symptoms, probably due to continuous surveillance of B-cell proliferation by immuno-regulatory T-cells. However, EBV is also involved in the pathogenesis of the aggressive monoclonal B-cell tumour, endemic Burkitt's lymphoma (eBL), implying that control of EBV-infected B-cell multiplication can break down under certain circumstances [1,2]. Epidemiological evidence suggests that malaria is a risk factor for eBL, as the geographical distributions of *Plasmodium falciparum *malaria and eBL strikingly overlap, and both diseases mainly affect children, who are also most susceptible to malaria in areas of intense *P. falciparum *transmission [3]. Furthermore, T-cells from healthy EBV-positive individuals inhibit spontaneous *in vitro *proliferation of autologous B-cells, whereas this does not happen in *P. falciparum *patients [4]. Finally, malaria is associated with marked polyclonal B-cell activation, which in turn increases the likelihood of malignant transformation of EBV-infected B-cells and subsequent development of eBL [5].

The nature and mechanisms of the T-cell restriction of EBV-infected B-cell proliferation remain only partly understood, not least because the majority of available studies pre-date detailed knowledge of the T-cell receptors and other functionally important markers of various T-cell subsets. More recently, marked differences have been reported in T-cell composition relating to *P. falciparum *exposure, and associated perturbations among children from endemic areas, who get malaria [6-8]. Thus, frequencies and absolute numbers of TCR-*γδ *cells, in particular cells expressing a T-cell receptor composed of a V*δ *1 chain and *γ*-chains other than V*γ*9, are several-fold higher in malaria-exposed than in non-exposed individuals, in whom such cells usually constitute a very minor subset (<1% of T-cells) [8]. Furthermore, V*δ*1^+ ^T-cells may transiently encompass more than a third of circulating T-cells and show a highly activated phenotype in children with *P. falciparum *malaria [8]. Although the function of V*δ *1^+ ^T-cells remains obscure, several studies have suggested that this subset is immuno-regulatory and controls multiplication of activated and EBV-transformed B-cells. On this basis, the present study was designed to investigate possible differences in T-cell subset composition and activation status in *P. falciparum-exposed *Ghanaian children with and without eBL. This study presents evidence of selective changes in numbers and activation status of TCR-*γδ*^+ ^cells in Ghanaian children with eBL, similar to *P. falciparum*-induced changes.

## Methods

### Patients

Included in the study were 21 patients (mean age: 7.0 years; range: 3–11 years) from all parts of Ghana who were referred to the Burkitt's Tumour Centre at Korle-Bu Teaching Hospital, Accra with a diagnosis of eBL on the basis of clinical information and cytological examination of tumour aspirates by a pathologist. In addition, 15 healthy children, matched for age and sex, from a nearby community (Dodowa town outside Accra) served as controls. Informed consent was obtained from all parents or guardians before their children were enrolled in the study. The Institutional Review Board at Noguchi Memorial Institute and the University of Ghana Medical School Scientific Research and Ethical Committee both granted ethical approvals for the study.

### Blood sample collection and processing

Venous blood samples were collected into sterile 10 ml heparinized vacutainer tubes using sterile butterfly needles. Peripheral blood mononuclear cells (PBMC) were isolated by Lymphoprep (Nycomed Pharma, As, Oslo) density gradient centrifugation. A volume of 5 ml of venous blood was carefully layered on top of 2 ml of Lymphoprep and centrifuged at 814 g for 30 minutes. The ring of PBMC was removed and washed three times in RPMI-1640 containing 10% heat-inactivated foetal calf serum (FCS) supplemented with gentamycin, and L-glutamine. Peripheral blood mononuclear cells (PBMC) were cryopreserved in liquid nitrogen as described [9].

### Parasitological and haematological examinations

An automated haematology analyzer (Sysmex KX-21, Japan) was used to determine all the 21 haematological parameters of the patients and the subjects. The absolute counts of lymphocytes were determined from this analysis. Each blood sample was also examined for presence of malaria parasites. Thick and thin blood smears were prepared, dried and the thin smears fixed in methanol. The films were then stained with freshly prepared 10% Giemsa (Laboratory Supplies, Poole BH15 ITD, England), washed carefully and thoroughly under running tap water. The slides were dried and observed with immersion oil under a light microscope (Olympus BH2, Japan) at 100× magnification, for the presence of *P. falciparum *parasites.

### Flow cytometry

On the day of analysis by flow cytometry, the PBMCs were quickly thawed in a water bath at 37°C, washed in PBS, adjusted to 1 × 10^6^/ml, and labeled with combinations of subset- or activation-specific monoclonal antibodies directly conjugated to fluorescein isothiocyanate (FITC), phycoerythrin (PE) or PE-Cy5. Antibodies used were directed against CD3 (UCHT1; DAKO, Glostrup, Denmark), CD4 (MT310; DAKO), CD8 (DK25; DAKO), CD25 (ACT-1; DAKO), CD69 (L78, Becton Dickinson (BD) Biosciences), CD95 (DX2; BD Immunocytometry Systems), HLA-DR (L243; BD Immunocytometry Systems), TCR-*γδ *(11F2; BD PharMingen (PE) and 11F2; BD Immunocytometry Systems (FITC), V*δ*1 (TS8.2; Endogen), V*δ*2 (B6; BD PharMingen), V*δ*3 (ImmunoTech, Marseilles, France), V*γ*9 (B3; BD PharMingen) and B-cell (FMC7; DAKO). The PBMC were subsequently washed once and a minimum of 8,000 forward-and side-scatter gated mononuclear cells analysed on a FACScan flow cytometer (Becton Dickinson, Japan).

### Statistical analysis

The statistical significance of differences in medians was calculated by the Mann-Whitney rank-sum test. Confidence intervals for median differences were calculated using CIA 2.0 software [10]. Values of P < 0.05 were considered significant.

## Results

### Frequency of T-cells is lower in eBL patients than in healthy controls

The mean frequency of peripheral blood CD3^+ ^cells was significantly lower in BL patients (66.5 [47.6 to 72.1] than in healthy controls (81.7 [76.4 to 86.4]), p < 0.001. This is mainly due to low TCR-*γδ*^+ ^cell numbers. In contrast, the proportions of CD4^+^cells, 63.5 [53.6 to 65.7] for eBL against 68.0 [57.8 to 75.0] for healthy controls, p = 0.11 or CD8^+ ^cells, 27.8 [26.6 to 31.7] for eBL against 20.9 [16.6 to 29.7] healthy controls, p = 0.08, were relatively unaffected. (Table 1), as were the mean numbers of peripheral blood mononuclear cells (eBL patients: 3.2 × 10^9^/L [95% confidence interval 2.4 to 4.0 × 10^9^/L], control donors: 3.4 × 10^9^/L; [2.7 to 4.0 × 10^9^/L]; student's t-test: P = 0.71).

**Table 1:** Lymphocyte subset frequencies (median percentages [95% confidence intervals]*) in Burkitt's lymphoma patients and healthy control donors

Subset		Healthy controls	eBL patients	Difference	P(T)*
CD3^+**^	-	81.7 [76.4 to 86.4]	66.5 [47.6 to 72.1]	-15.4 [-34.1 to -7.5]	<0.001
CD19^+**^	-	0.4 [0.2 to 1.0]	5.8 [4.8 to 8.8]	5.4 [4.2 to 8.4]	<0.001
CD3^+^	CD4^+^	63.5 [53.6 to 65.7]	68.0 [57.8 to 75.0]	5.0 [-1.9 to 13.1]	0.11
CD3^+^	CD8^+^	27.8 [26.6 to 31.7]	20.9 [16.6 to 29.7]	-6.0 [-11. 5 to 1.8]	0.08
CD3^+^	TCR-*γδ*^+^	7.5 [4.9 to 15.5]	3.3 [2.6 to 5.4]	-3.6 [-7.9 to -1.2]	0.006
TCR-*γδ*^+^	V*γ*9^+^	45.2 [34.8 to 59.8]	18.8 [14.2 to 29.8]	-25.2 [-35.4 to -15.1]	<0.001
TCR-*γδ*^+^	V *δ*2^+^	32.3 [12.3 to 45.1]	9.2 [6.9 to 13.9]	-2 1.2 [-3 1.2 to -4.8]	0.007
TCR-*γδ*^+^	V *δ*3^+^	7.7 [5.0 to 11. 7]	9.8 [5.4 to 14.3]	2.2 [-1.9 to 6.6]	0.25
TCR-*γδ*^+^	CD4^+^	4.8 [3.7 to 9.6]	7.5 [4.9 to 34.8]	2.9 [0.1 to 24.2]	0.04
TCR-*γδ*^+^	CD8^+^	31.2 [26.4 to 48.3]	36.6 [26.0 to 45.9]	0.0 [-8.0 to 11.0]	0.98

* The statistical significance of differences in medians was calculated by the Mann-Whitney rank-sum test. Confidence intervals for median differences were calculated using CIA 2.0 software [1].

** Of all lymphocytes identified by forward and side scatter properties

### Activation status of T-cells in eBL patients

The frequencies of TCR-*γδ*^+ ^CD25 and HLA-DR^+ ^were significantly higher in eBL patients compared to healthy controls, p = 0.007 and 0.003 respectively. There were no differences between the two groups with respect to TCR-*γδ*^+ ^CD69^+ ^and TCR-*γδ*^+ ^CD95^+ ^(Table 2). With respect to TCR-*γδ*^+ ^cells, the frequency of CD25^+ ^cells was lower in eBL patients (8.0 [4.9 to 11.1]) compared to the healthy controls (13.5 [9.3 to 16.9]), p = 0.04.

**Table 2:** Frequencies (median percentages [95% confidence intervals]*) of activated cells within lymphocyte subsets in Burkitt's lymphoma patients and healthy control donors

Subset		Healthy controls	BL patients	Difference	P(T)*
TCR-*αβ*^+**^	CD25^+^	13.5 [9.3 to 16.9]	8.0 [4.9 to 11.1]	-5.0 [-8.6 to -0.6]	0.04
TCR-*αβ*^+^	CD69^+^	4.1 [1.2 to 8.5]	2.2 [1.1 to 4.6]	-1.2 [-4.2 to 0.8]	0.20
TCR-*αβ*^+^	HLA-DR +	12.6 [7.6 to 21.1]	20.2 [11. 4 to 30.0]	7.2 [-0.6 to 16.9]	0.07
TCR-*αβ*^+^	CD95^+^	36.7 [29.1 to 44.8]	45.4 [16.2 to 58.4]	6.2 [-12.2 to 20.6]	0.40
TCR-*γδ*^+^	CD25^+^	1.6 [0.9 to 4.6]	5.8 [2.6 to 11. 9]	3.6 [1.0 to 8.1]	0.007
TCR-*γδ*^+^	CD69^+^	7.5 [2.0 to 14.6]	8.9 [4.0 to 13.3]	1.4 [-4.0 to 8.1]	0.61
TCR-*γδ*^+^	HLA-DR +	33.3 [16.7 to 50.0]	54.8 [39.6 to 67.8]	22.7 [8.8 to 36.2]	0.003
TCR-*γδ*^+^	CD95^+^	42.5 [32.5 to 58.3]	68.8 [30.4 to 89.0]	21.3 [-1.7 to 41.0]	0.10

* The statistical significance of differences in medians was calculated by the Mann-Whitney rank-sum test. Confidence intervals for median differences were calculated using CIA 2.0 software [1].

** Defined as CD3^+ ^TCR-*γδ*-negative cells

## Discussion

In a previous study, it was reported that frequencies of TCR-*γδ*^+ ^cells are selectively increased in *P. falciparum*-exposed healthy Ghanaians in general, and in children in particular [7]. The findings regarding the healthy children included in the present study are in agreement with the earlier data (Table 1, left). It has been have further reported that episodes of *P. falciparum *malaria among such children led to marked perturbations in lymphocyte subset composition, which particularly affected TCR-*γδ*^+ ^cells [6,8]. Interestingly, the children with eBL studied here showed changes in lymphocyte composition that resemble the changes seen in children with *P. falciparum *malaria (Table 1). Thus, a marked reduction in the proportion of CD3^+ ^cells in eBL patients was observed, a difference which appeared to be mainly due to perturbations among TCR-*γδ*^+ ^cells (Table 1). In contrast, the proportions of CD4^+ ^or CD8^+ ^cells were relatively unaffected (Table 1). The mean numbers of peripheral blood mononuclear cells were also unaffected (eBL patients: 3.2 × 10^9^/L [95% confidence interval 2.4 to 4.0 × 10^9^/L], control donors: 3.4 × 10^9^/L; [2.7 to 4.0 × 10^9^/L]; student's t-test: P = 0.71).

In children with *P. falciparum *malaria, the selective depletion of T-cells and especially of TCR-*γδ*^+ ^cells [6,11] appears to be due to the a transient redistribution of activated T-cells to sites of endothelial inflammation [12,13]. Whether the changes observed here have the same aetiology as in malaria patients remains speculative. However, the activation phenotype of the T-cells obtained from the eBL patients (Table 2) points to selective reallocation of T-cells – and in particular of TCR-*γδ*^+ ^cells – as an important factor behind the observed changes in lymphocyte subset composition in eBL patients (Table 1). Previous findings of increased serum levels of soluble IL-2 receptor (CD25) and soluble ICAM-1 (CD54) in eBL patients [14,15], which again resembles the situation *in P. falciparum *malaria [16], lends further support to this eBL reallocation hypothesis.

Within the TCR-*γδ*^+ ^cell compartment, the perturbations in relation to *P. falciparum *malaria among children living in endemic areas are mainly caused by a dramatic increase in the proportion of *γδ *T-cells expressing a T-cell receptor composed of a V*δ*1 chain in combination with *γ*-chains other than V*γ*9 [8]. Although V*δ*1^+ ^cells were not directly examined here, the pronounced reduction in the proportions of the complementary V*γ*9^+ ^and V*δ*2^+ ^subsets in eBL patients (Table 1) constitute indirect evidence that the proportion of V*δ*1^+ ^cells is increased in eBL patients as it is in children with *P. falciparum *malaria.

Taken together, the data presented here show a remarkably similar impact on peripheral lymphocyte composition of eBL and *P. falciparum *malaria, which may reflect a causal link between the two diseases. The characteristic massive B-cell activation in *P. falciparum *malaria [17,18] appears to compromise homeostatic T-cell control of proliferation of EBV-immortalized B-cells [19]. It is tempting to speculate that these regulatory T-cells may include V*δ*1^+ ^cells, as it has been shown that T-cells belonging to this subset can recognize and kill activated B-cells [20-22].

## Conclusion

Malaria-induced B-cell activation may thus precipitate malignant transformation of EBV-infected B-cells. Incomplete elimination of malignant T-cells due to defects in auto-regulatory T-cells may subsequently lead to eBL. This study provides insights regarding the pathogenesis of eBL and suggests a plausible link between the pathogenesis of *P. falciparum *malaria and eBL.

## Authors' contributions

GF: designed the study, carried out laboratory work, data analysis and interpretation and drafted the manuscript.

JW assisted with design of study, selection, clinical examination and management of BL patients, as well as revision of manuscript for intellectual content.

J KAT assisted with flow cytometry and data analysis.

LH designed the study, carried out data analysis and extensive revision of manuscript for intellectual content

BDA designed study, assisted with data analysis and extensively revised manuscript for intellectual content.
